# Organic Solar Cells: Understanding the Role of Förster Resonance Energy Transfer

**DOI:** 10.3390/ijms131217019

**Published:** 2012-12-12

**Authors:** Krishna Feron, Warwick J. Belcher, Christopher J. Fell, Paul C. Dastoor

**Affiliations:** 1 CSIRO Energy Technology, PO Box 330, Newcastle, NSW 2304, Australia; E-Mails: warwick.belcher@newcastle.edu.au (W.J.B.); paul.dastoor@newcastle.edu.au (P.C.D.); 2 Centre for Organic Electronics, University of Newcastle, Callaghan, NSW 2308, Australia; E-Mail: chris.fell@csiro.au

**Keywords:** organic solar cells, photovoltaic, exciton, FRET, energy transfer, photoconversion mechanism, review

## Abstract

Organic solar cells have the potential to become a low-cost sustainable energy source. Understanding the photoconversion mechanism is key to the design of efficient organic solar cells. In this review, we discuss the processes involved in the photo-electron conversion mechanism, which may be subdivided into exciton harvesting, exciton transport, exciton dissociation, charge transport and extraction stages. In particular, we focus on the role of energy transfer as described by Förster resonance energy transfer (FRET) theory in the photoconversion mechanism. FRET plays a major role in exciton transport, harvesting and dissociation. The spectral absorption range of organic solar cells may be extended using sensitizers that efficiently transfer absorbed energy to the photoactive materials. The limitations of Förster theory to accurately calculate energy transfer rates are discussed. Energy transfer is the first step of an efficient two-step exciton dissociation process and may also be used to preferentially transport excitons to the heterointerface, where efficient exciton dissociation may occur. However, FRET also competes with charge transfer at the heterointerface turning it in a potential loss mechanism. An energy cascade comprising both energy transfer and charge transfer may aid in separating charges and is briefly discussed. Considering the extent to which the photo-electron conversion efficiency is governed by energy transfer, optimisation of this process offers the prospect of improved organic photovoltaic performance and thus aids in realising the potential of organic solar cells.

## 1. Introduction

Organic solar cells have received considerable interest over the last decade as they offer the potential for cheap renewable energy through direct photo-electron conversion of abundantly available sun light [1]. Organic solar cells are thin, lightweight and flexible, which makes them easy to implement and useable for a variety of purposes. Rapid progress has been made in extending operating lifetimes and improving power conversion efficiency (PCE), which has reached 10 % with small scale devices [2]. The efficiency of OPV devices has systematically increased over the last decades [3] as a result of progress made in understanding the photoconversion mechanism, which instructs device design and material synthesis [4].

**Figure 1:**
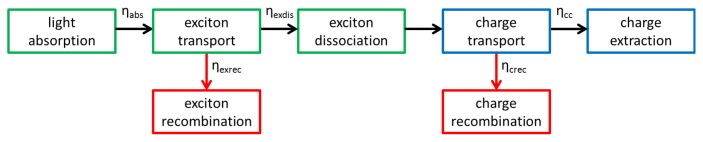
Overview of the photoconversion mechanism in organic solar cells. Processes that involve FRET are indicated in green and recombination pathways in red.

The photoconversion mechanism may be divided into five components (see Figure 1): light absorption, exciton transport, exciton dissociation, charge transport and charge extraction. The latter two are often captured by a single charge collection term [5]. The overall quantum efficiency, *η*, is determined by the efficiency of these processes through

(1) $$\eta =\eta_{abs}\eta_{exdis}\eta_{cc}$$

where *η_abs_* is the light absorption efficiency, *η_exdis_* the exciton dissociation efficiency (includes exciton transport) and *η_cc_* the charge collection efficiency.

### 1.1. Light Absorption

The photoactive component of organic photovoltaic (OPV) devices consists of conjugated (alternating double and single bonds) carbon based materials [6]. The conjugated aspect together with a low dielectric constant (*ɛ_r_* ~ 2 − 4) gives rise to a different photo conversion mechanism than commonly found in inorganic solar cells [7]. Whereas free charge carriers are created directly in inorganic solar cells, bound electron-hole pairs (or excitons) are created upon light absorption in OPV devices [8]. As such, the exciton population is directly related to the number of absorbed photons [9]. These excitons remain bound in organic electronic materials due to Coulomb attraction and the localised energy nature of organic semiconductors [10]. Photons with energy larger than the bandgap will yield excitons of the same energy, since excited electrons rapidly relax to the lowest unoccupied molecular orbital (LUMO) level [11]. This process occurs on a femtosecond timescale [12] and is much faster than competing processes such as exciton hopping or recombination [13]. Organic semiconductors generally have high absorption coefficients at the peak of their absorption spectrum [14,15] and, as such, the exciton generation rate can be maximised by increasing the overlap of the absorption profile and AM1.5 solar spectrum [16]. Consequently, substantial efforts have been focused on developing low band gap polymers [17,18] to extend the spectral response of OPV devices.

### 1.2. Exciton Transport

Exciton transport is governed by diffusion processes, with transport directed away from regions of high exciton concentration [19]. The lack of molecular interaction in organic semiconductors influences exciton transport, which occurs through a hopping mechanism [10]. If an exciton is not separated within its lifetime, the electron-hole pair simply recombines and is lost. Organic semiconductors generally exhibit small exciton diffusion lengths on the order of 10 nm [19–23] and thus the range over which dissociation must occur is highly restricted in these materials [24,25].

### 1.3. Exciton Dissociation

A typical exciton binding energy is of the order of 0.25–1 eV [7] and is the minimum energy required to dissociate the exciton. Thermal and electrical (electric field) energies are therefore insufficient under normal operating conditions to dissociate the exciton, and instead the chemical potential difference between two organic semiconductors is utilised [26]. At the interface between these two semiconductors (heterointerface), electron transfer occurs from the electron donor to the electron acceptor with near unity efficiency (provided that the LUMO offset and/or HOMO offset is favourable) [8], while the hole remains in the hole conducting moiety, thus structurally separating the electron and hole. Hole transfer from the electron acceptor into the electron donor may also occur and also yields exciton dissociation. Impurities may also induce dissociation [27], but this process often leads to trapped charges instead of free charge carriers and is thus detrimental to device performance [7]. The heterointerface is the main dissociation site in the photoactive layer and dissociation at this interface is often treated as the only light induced charge generation mechanism [28].

The exciton dissociation efficiency is also a function of the heterointerface area and the ability of the exciton to diffuse to this interface [5]. To maximize exciton dissociation, a large heterointerface is needed. This large heterointerface is commonly achieved using so-called bulk-heterojunction (BHJ) morphologies. Generally, both organic components are mixed in a common solvent and after the deposition of this solution and subsequent annealing treatments, an optimal structure may be achieved with an average domain size on the order of the exciton diffusion length [29]. Once the exciton is dissociated, the electron and hole still experience significant Coulomb attraction and although structurally separated, they remain in a bound state with a finite lifetime [30]. This bound state is commonly referred to as a charge-transfer (CT) state [31], but is also known as the geminate pair, bound radical ion pair, polaron-polaron pair, charge-transfer excitons or exciplex state [31–37]. Coulomb interaction remains significant until the electron-hole separation distance exceeds the Coulomb capture radius (also known as the Onsager radius), which is defined as the radius at which Coulomb attraction equals thermal energy, *k_B_T*[24,31,38].

### 1.4. Charge Transport

Under the influence of the electric field (both the built-in field due to work function difference of electrodes and the externally applied field), Coulomb interaction, energetic disorder and morphology, charge carriers are transported through their associated transport networks (*i.e*., holes and electrons hop through the p-type and n-type material respectively). Electrons and holes may recombine and be lost or hop towards the electrodes where they can be extracted to yield a photocurrent [8].

In recent years, a number of reviews have been published that focus on specific areas of organic solar cells, for example, small molecules [1], polymers [5], large scale deposition techniques [39], economical aspects [6,40], low band gap polymers in general [17,18], specific low band gap material systems [41], tandem solar cells [11,21], charge photogeneration [31] and stability and degradation [42].

In this review, we discuss the latest models of the photo conversion mechanism in organic solar cells and focus on the role that Förster resonance energy transfer (FRET) theory plays in exciton harvesting, transport and dissociation. First we introduce FRET in the context of exciton transport. Then more advanced concepts that utilise FRET are discussed in the context of exciton harvesting and dissociation. Charge transport and in particular charge transfer dynamics are also discussed. FRET does not play a role in these processes, but they are required to understand the concept of an energy cascade, where FRET does have a significant role. Finally charge extraction is briefly discussed to provide a complete overview of the entire photoconversion mechanism, followed by concluding remarks.

## 2. Exciton Transport

Exciton transport plays an important role in the overall exciton dissociation process [24,43] and must be optimised to yield efficient OPV devices. Exciton diffusion is often modelled using FRET. In 1948 Förster published a quantum-mechanical treatment of transfer of electronic excitation energy between similar molecules (oscillators) [44]. These oscillators effectively behave like dipoles and their Coulomb interaction governs the energy transfer rate. Since energy must be conserved, the acceptor molecule must have energy states available such that the gain in energy of the acceptor is equal to the loss in energy of the donor molecule. The available energy states can be determined experimentally by measuring the absorption spectrum and fluorescence spectrum of the acceptor and donor species. Förster determined that the energy transfer rate, *k_FRET_*, is given by

(2) $$k_{FRET}=\frac{1}{\tau_{0}}\;{\left(\frac{R_{0}}{r}\right)}^{6}$$

where *τ*_0_ is the fluorescence lifetime of the exciton, *r* is the distance between donor and acceptor, and *R*_0_ is the Förster radius, which is the critical distance at which the fluorescence probability is equal to the energy transfer probability and is given by

(3) $$R_{0}^{6}=\frac{9000\;\text{ln }(10)\;\varphi \kappa^{2}}{128\pi^{5}N_{A}n^{4}}\int_{0}^{\infty }ɛ\;(\nu )\;F(\nu )\frac{d\nu }{\nu^{4}}$$

where *κ* is the orientation factor between donor and acceptor, *ϕ* is the fluorescence quantum yield, *n* is the refractive index, *N_A_* is Avogadro’s number, *ν* is the frequency of light, *ɛ* is the peak-normalised fluorescence spectrum of the donor and *F* is the absorption spectrum of the acceptor. The integral is referred to as the overlap integral and quantifies the dipole-dipole interaction of donor and acceptor.

Equations (2) and (3) have been successfully used in many organic semiconductors [20] where the donor and acceptor molecules can be approximated by point dipoles, which is largely valid for hopping within a single material. However, intermolecular transfer between different species is not well modelled by the point-dipole approximation [45]. In addition, for polymers such as polythiophenes, the point-dipole approximation fails and a line-dipole approximation must be used in calculating intermolecular transition rates [46]. In the line-dipole approximation, the transition dipole moments are distributed along the polymer backbone and their interaction is integrated over all infinitesimal dipole moments (replacing the integral in Equation (3)). In general, the line-dipole approximation should be used when the conjugation length is much larger than the separation between molecules [47]. Other quantum chemical-methods such as the transition density cube method [48] and the distributed monopole approximation [49] calculate the Coulombic coupling between transition states more realistically, but are computationally intensive [46,50]. Energy transfer is usually much faster than intramolecular vibrational relaxation, which means that the donor and acceptor sites are “frozen” while energy transfer occurs [51]. However, there are some exceptions [52,53] in which case a generalised version of Förster theory is more appropriate [54]. Other complications that preclude the use of the point-dipole model are energetic and positional disorder [55].

**Figure 2:**
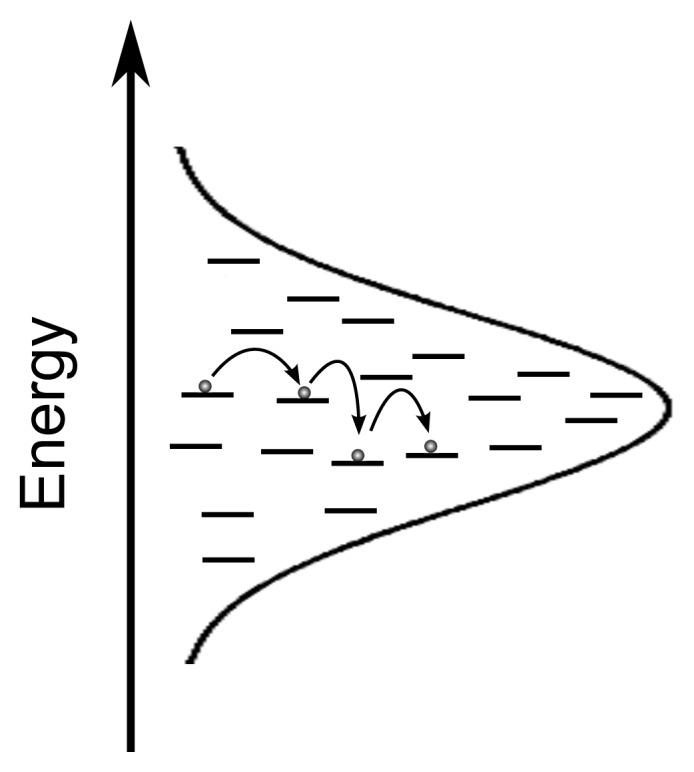
Exciton hopping in the intrinsic DOS which is approximated by a Gaussian distribution.

The simplest version of FRET theory (Equation (2)) does not consider effects of inhomogeneous broadening and energetic disorder, which sometimes leads to considerable prediction errors of exciton diffusion parameters [55,56]. Exciton transport in organic semiconductors can be modelled as a hopping process in which charges move from donor to acceptor governed by the availability of empty states in the density-of-states (DOS), which is often described by a Gaussian distribution with standard deviation, *σ*[57–59]. In Monte Carlo models of exciton transport, energetic disorder of the available empty energy states is taken into account by assigning energy levels according to this Gaussian distribution as depicted in Figure 2.

Consequently, the hopping rates are frequently calculated using:

(4) $$k_{FRET}=\frac{1}{\tau_{0}}\;{\left(\frac{R_{0}}{r}\right)}^{6}\;f\;(E_{i},E_{j})$$

where the *f*-function is the Boltzmann factor and considers the relative energy state differences of donor and acceptor according to the relationship:

(5) $$f\;(E_{i},E_{j})=\{\begin{array}{ll} e^{-\frac{E_{j}-E_{i}}{k_{B}T}} & E_{j}>E_{i}\\ 1 & E_{j}<E_{i} \end{array}$$

where *E_i_* and *E_j_* is the energy of the donor and acceptor respectively. Alternatively, the energy dependence may be more accurately assessed using the overlap integral in Equation (3) [60].

Exciton transport may be heavily influenced by energetic disorder of the intrinsic DOS. Energetic disorder is usually quantified by the standard deviation of the Gaussian distribution, *σ*. As this disorder increases, the exciton diffusion length will decrease considerably. The diffusion length, *L*, is defined as

(6) $$L^{2}=\langle r^{2}\rangle$$

where *r* is the distance between the location of exciton creation and exciton annihilation. Förster derived how the diffusion constant, *D*, relates to the Förster radius through [44]

(7) $$D=\frac{s}{t_{0}}{\left(Nc\right)}^{4/3}\;R_{0}^{6}$$

where *N* is the number of chromophores per millimole, *c* is the chromophore concentration and *s* is a constant that relates to the molecular distribution of the molecules. It immediately follows from Equation (7) and the relation between *D* and *L* that a cubic relation exists between *L* and *R*_0_[61]

(8) $$L=\sqrt{6Dt_{0}}=\sqrt{6s{\left(Nc\right)}^{4/3}}R_{0}^{3}$$

**Figure 3:**
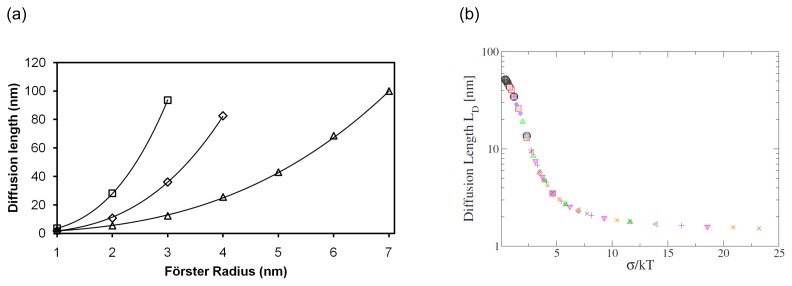
(**a**) *L* as a function of *R*_0_ for *σ* = 0 eV (squares), *σ* = 0.05 eV (diamonds) and *σ* = 0.09 eV (triangles). Reproduced with permission from [24]. Copyright 2012 by The American Institute of Physics; (**b**) Diffusion length (*L_D_*) as a function of *σ*. Reprinted with permission from [62]. Copyright 2009 by The American Physical Society.

For large values of *σ*, this cubic relation still holds but *L* decreases considerably as shown in Figure 3(a) and *L* becomes relatively insensitive to *R*_0_[62]. Figure 3b shows the dramatic decrease in *L* as *σ* increases. As the exciton relaxes, it becomes more difficult to overcome energy barriers. It has been shown experimentally that excitons in lower lying energy states have a much lower probability of energy transfer to states of higher energy [63] as would be expected from Equation (5). Thermal energy also influences the ability to overcome local energy barriers, which is why *σ* should be discussed in relation to kT as is done in Figure 3b. Monte Carlo modelling suggests that at high values of *σ* (up to 0.07 eV was investigated), *L* is more sensitive to temperature (300 K–450 K), since thermal energy aids in overcoming energy barriers [59]. Energetic disorder is both an inherent material property as well as a result of structural order [64]. In many systems, disorder (either energetic or structural) may be the most important diffusion length limiting factor [65]. As a result, thermally sublimed structurally ordered materials often exhibit larger diffusion lengths than less ordered spin-cast polymers [19,66,67]. Structural order not only affects the spatially averaged diffusion length, but may also affect the preferred direction of exciton hops as indicated by Monte Carlo simulations [51,59,68]. Hence, molecular ordering could be used to direct excitons to the heterointerface where efficient exciton dissociation occurs.

Exciton transport has been modelled using a simple random walk, which does not take into account the energetic landscape or non-nearest neighbour hops [24]. The term random walk could also refer to models where the step size is not constant or a biased random walk model where the energetic landscape is taken into account. Here we specifically compare a simple random walk, *i.e*., a non-biased random walk with a constant step size (only nearest neighbours are considered) and where energy related processes are not considered. The advantage of this approach is that it is a simple model to implement from both a computational and conceptual point of view. However, as can be inferred from Equation (5), depending on the temperature and energetic disorder, excitons are likely to hop to low energy sites in the semiconductor [24,56]. As such, at the point of dissociation, excitons modelled using the FRET method in combination with Equation (5) will on average reside in a lower energy state compared with excitons modelled using a simple random walk approach. The energy at dissociation is of importance as the created charge carriers will also lie in lower energy states. The result is that, on average, more charge carriers in the FRET model have to hop energetically upwards, which is less probable and, consequently, the simple random walk model overestimates charge separation. In addition, a simple random walk does not consider transfer to non-nearest neighbour sites (long-range exciton transfer), which may be important for very small BHJ domain sizes. Nonetheless, exciton diffusion has been successfully modelled using random walk approaches [21,23,69]. Indeed, recent work has shown that modelling exciton transport using a simple random walk for BHJ morphologies with standard domain sizes introduces an error of only 2% in simulated current [24].

Photoluminescence (PL) measurements are frequently used to measure exciton transport properties, such as *L* or *R*_0_. In these experiments, single component layers are fabricated in order to reduce complexity and independently determine *L* for the material of interest. Some of the variables in Equation (3) are difficult to measure directly and consequently *R*_0_ is often treated as a fitting parameter since it can be easily determined in independent PL experiments [21,23,70].

**Figure 4:**
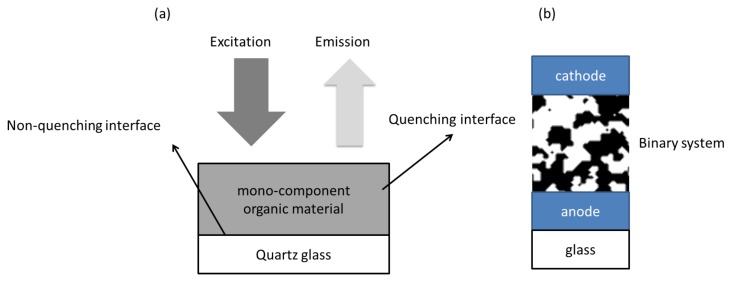
A typical sample for (**a**) single material PL measurements and (**b**) OPV devices.

In PL experiments where a single component layer is covered by a quenching layer, such as titania or simply air (molecular oxygen is known to be an efficient collisional quencher [71]), exciton transfer within the single component system can be approximated with a simple random walk (*i.e*., considering nearest neighbour hops only) [72–74]. However, it has been shown that when using quenchers that allow for energy transfer as opposed to collisional quenching only, Förster transfer must be considered for an accurate determination of the diffusion length [22,75]. In addition, for the bi-component blend material that comprises the active layer of BHJ OPV devices (Figure 4) a non-continuous finely mixed system may form in which long-range energy transfer may have a significant effect on exciton transport and thus exciton dissociation. Thus, for OPV devices Förster theory should be used to model exciton diffusion since it considers long-range energy transfer in addition to purely diffusive transport and thus avoids an overestimation of the diffusion length, especially for small values of *L*[22,76,77].

**Figure 5:**
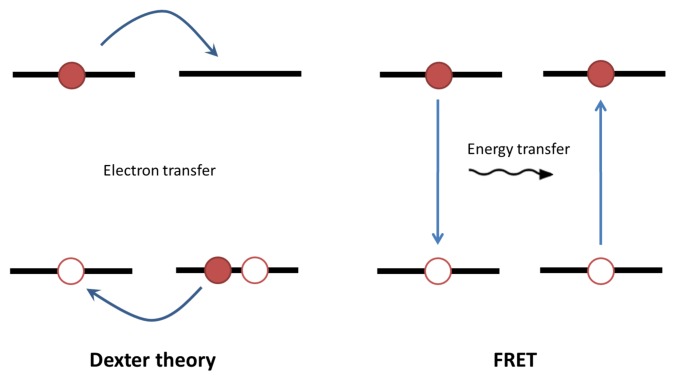
**Left**: exciton transport through charge transfer. **Right**: exciton transport through energy transfer.

Usually, only singlet excitons can undergo Förster energy transfer and generally triplet exciton transport more accurately follows Dexter theory. Dexter’s approach treats exciton transport as electron transfer as opposed to energy transfer and predicts that the exciton transfer rate decays exponentially with distance. However, when a triplet exciton is located near a phosphorescent organometallic donor, it has been shown it may undergo Förster transfer [78]. Figure 5 depicts the fundamental difference between the Dexter and FRET mechanism for exciton transport.

Singlet excitons have short lifetimes on the order of nanoseconds [66,79–81] while triplet excitons have lifetimes on the order of microseconds [21,43,82,83]. While triplet excitons tend to have longer lifetimes, they also tend to have lower diffusivities (as predicted by the difference in transfer rates calculated from FRET and Dexter theory) and thus travel slower, which results in similar diffusion lengths for both types of excitons [20].

In contrast to excitons that are created through charge injection, light absorption in organic semiconductors yields only singlet excitons and thus triplet excitons can only be formed through intersystem crossing [84,85]. However, these triplet excitons slowly decay to the ground state since they lie in an energy state that is too low for dissociation [85]. Thus, intersystem crossing does not generally contribute to charge generation and may be viewed as a (delayed) recombination mechanism. There are two exceptions to this statement: upconversion [86] and singlet fission based OPV devices. Photochemical upconversion is achieved by utilising a sensitizer with efficient singlet-triplet intersystem crossing. Triplet excitons are passed on to a second species (referred to as the emitter [87] or acceptor/annihilator), where two triplet excitons annihilate to form a singlet exciton with more energy than the original singlet exciton generated in the sensitizer. The resulting high energy singlet exciton may be emitted and absorbed by photoactive materials. Upconversion can thus be utilized to absorb light in the high wavelength region where common OPV materials do not absorb well. Upconversion is an advanced concept and as of yet only insignificant solar cell enhancements are obtained, but future improvements are expected [87]. Singlet fission is the process of converting a single singlet exciton into two triplet excitons through coupling with a nearby molecule in the ground state [88]. As a consequence, the number of extracted charges (*i.e*., photocurrent) could potentially be doubled, while halving the maximum achievable voltage as the triplet excitons have at most half the energy of the singlet exciton. Quantum efficiencies have been shown to increase by 45% [89], but as of yet the PCE of systems that use this advanced concept remain low [90,91].

Notwithstanding these exceptions, intersystem crossing is irrelevant for certain material systems, including the commonly used P3HT:PCBM system, as the overall rate covering singlet diffusion and subsequent exciton dissociation at the hetero-interface is much higher than the intersystem crossing rate [92]. Consequently, singlet excitons are the dominant exciton species and thus FRET is the preferred theory to describe exciton behaviour in organic solar cells.

## 3. Exciton Harvesting

Organic semiconductors generally exhibit high absorption coefficients, but only for a narrow wavelength region [15]. In order to increase the spectral range of absorption, chromophores (sensitizers) that give photoluminescence in the absorption region of the active materials in the solar cell may be utilised [93]. With a dedicated light harvesting layer, thinner active layers (active in exciton dissociation and charge transport) can be used without compromising light absorption. Thinner layers are known to exhibit better internal quantum efficiency [94] and thus high overall efficiency may be achieved.

**Figure 6:**
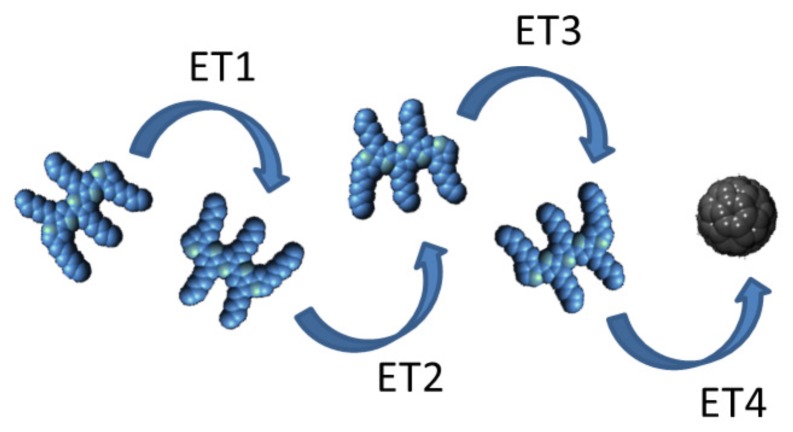
Exciton transport within a single semiconductor (ET1, ET2, ET3) followed by inter-species donor-acceptor energy transfer (ET4).

FRET is not only used to describe exciton transport within a single semiconductor phase, but is often used to characterise energy transfer between different species of molecules [95]. The rate of energy transfer, *k_ET_*, not only depends on the overlap integral, but also on the donor-acceptor orientation (Equation (3)), which in turn depends on morphology and may be influenced by annealing treatments and moiety fraction [96]. *k_ET_* is readily determined from time resolved photoluminescence decay measurements as it is given by [63]:

(9) $$k_{ET}(t)=-\frac{d}{dt}\text{ln }\left(\frac{p(t)}{a(t)}\right)$$

where *p*(*t*) and *a*(*t*) are the photoluminescence of the donor in the absence and presence of the acceptor respectively. The overall energy transfer efficiency must also take into account the transition rates of competing processes such as exciton diffusion to annihilation states (e.g., energy traps) and natural inherent exciton decay [95]. A donor-acceptor system for energy transfer includes all the complexities of exciton transport in a homogenous layer plus the added step of energy transfer between two different materials, which makes optimisation a non-trivial task. One way of assessing the impact of intra-species transport in the donor in relation to the final donor-acceptor transition step (ET4 in Figure 6) is by measuring time dependent photoluminescence and fitting a stretched-exponential of the form

(10) $$I(t)=I_{0}e^{-{\left(kt\right)}^{\alpha }}$$

where *I*_0_ is the intensity at time *t* = 0 and *k* is the decay rate constant. *α* indicates the dimensionality and diffusion dynamics in the density of states of energy transfer [97]. For single step three dimensional FRET, *α* = 1*/*2, for single step two-dimensional FRET *α* = 1*/*3 [98] and for diffusive transport only (followed by a final nearest neighbour hop into the acceptor) *α* = 1 for a simple random walk. In the case of diffusive transport in an inhomogenously broadened density of states [95]*α* is slightly less than 1 since diffusion slows down with time due to energy relaxation [99].

**Figure 7:**
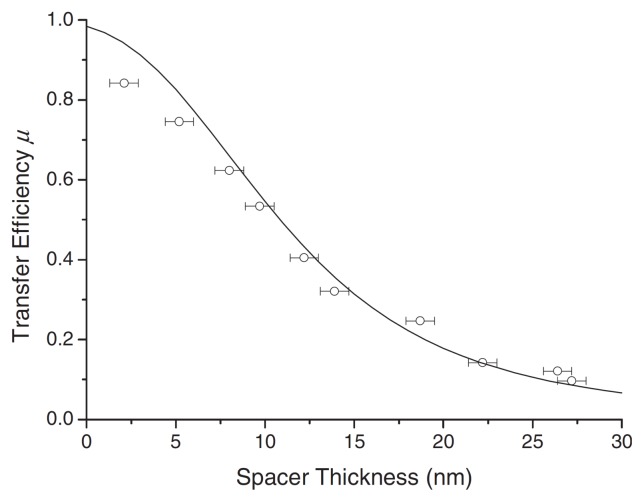
Energy transfer efficiency as a function of spacer thickness for a PFOBT–P3HT system. Reprinted with permission from [101]. Copyright 2008 by The American Physical Society.

When the overlap of the emission spectrum of the dye and the absorption spectrum of the absorbing photoactive material is considerable, as is the case for example for poly[9,9-dioctylfluorenyl-2,7-diyl)- co-(1,4-benzo-2,1′,3-thiadiazole)] (PFOBT) and P3HT, efficient energy transfer occurs for distances on the order of the domain size in BHJ devices [100], as is shown in Figure 7. A good fit for most of the curve is obtained by applying the point-dipole approximation (Equations (2) and (3)). However, for small distances the point-dipole approximation does not hold anymore and the curve starts to deviate from the measured results. In general, for intermolecular transfer between different species the point-dipole approximation should be used with caution at very short donor-acceptor distances and more advanced quantum chemical calculations may be required in certain cases [45].

While the concept of sensitising dyes was introduced in the context of OPV several years ago [76,93,100], only in the last couple of years has it been experimentally shown to increase PCE [102]. Materials with specific absorption and fluorescence spectra and high fluorescence yield are necessary and continue to be developed [102,103].

In order to improve the absorption range overlap with the AM1.5 spectrum, low or reduced band gap polymers have been heavily investigated [17]. Fullerenes exhibit excellent electron accepting and transporting properties, however, their ability to absorb visible light is limited. Consequently, the combination of a narrow low band gap polymer with common electron acceptors (*i.e*., fullerene derivatives) typically results in poor absorption in the 400–700 nm wavelength region. Thus, a significant amount of light is lost. In fact, the common standard PCBM:P3HT device absorbs mainly in this region and efficiencies of *~*4% is often achieved with this system [104]. Given that only 13% of the short-circuit current originates directly from PCBM light absorption [105], conceptually, PCE may be significantly increased if this lost absorption region is efficiently recovered.

Preliminary investigations have been conducted to demonstrate the potential of using a PCBM sensitising material [75,103]. Dyes with a band gap much less than 1.8 eV will not efficiently transfer energy to PCBM, as PCBM absorbs poorly in this region. Potential materials have been identified, but an experimental demonstration of improved PV performance as a result of PCBM sensitisation has only recently been demonstrated. Hesse *et al*. [102] successfully used perylene-diimid (PDI) as the PCBM sensitising agent in a bilayer device consisting of PCBM and a near UV absorbing small molecule, hexa-peri-hexabenzocoronene (HBC). The absorption spectrum of PDI covers the gap the between the absorption peaks of a low bandgap polymer such as poly[2,1,3-benzothiadiazole-4,7-diyl[4,4-bis(2- ethylhexyl)-4*H*-cyclopenta[2,1-b:3,4-b′]dithiophene-2,6-diyl]] (PCPDTBT) and PCBM and extends the absorption range of a PCBM:HBC device. External quantum efficiency spectra and photoluminescence measurements indicate that energy is transferred from PDI to PCBM. However, PDI does not only act as a sensitizer, as bilayer devices with PDI and no PCBM also yield working devices and thus PDI possesses photovoltaic properties in combination with HBC. These PDI:HBC devices perform poorer than the corresponding PCBM:HBC bilayer devices. Performance was maximised when both PDI and PCBM was used with HBC at a PDI/PCBM loading ratio of 50%. A power conversion efficiency of 0.4% was achieved, which is 60% more than the bilayer without PDI sensitisation. The concept has yet to be successfully implemented in high efficiency OPV systems.

Introducing a third sensitising component to the active layer, which already consists of an electron donating and accepting material, may be a viable way to enhance OPV performance, but also adds another layer of complexity with regards to morphology optimisation, as the sensitising component may disrupt the sensitive network between electron conducting and hole conducting moieties. The increased complexity of a third component makes it more difficult to understand recombination processes at all the interfaces [106].

In general, energy transfer is crucial in extending the absorption spectrum of OPV devices. However, dyes may also directly transfer the electron to the electron conducting moiety and holes to the hole conducting moiety [107,108]. Consequently, the electronic energy levels of the third photoactive component with respect to the other photoactive components and electrodes are of importance as well. Energy level mismatch in ternary blends has been shown to result in considerable recombination [109]. Simultaneous electron and hole transfer to the electron acceptor and donor materials respectively is only possible if the dye is in contact with both materials. The latter requirement results in a more restrictive morphology and is challenging to achieve unless the ternary systems naturally orders in such a way that the dye effectively forms at the heterointerface [110]. A ternary system could also form a mixture of two binary systems in parallel [111], essentially resulting in a single layer tandem cell.

Finally, another approach to increase the fraction of absorbed photons that does not involve direct contact of the absorbing dye with the PV active materials is the use of luminescent concentrators that luminesce at the desired wavelength and guide light to the PV materials through a waveguiding structure [93]. The waveguiding structure guides light parallel to the OPV devices, which may then be captured with a cell that is positioned side-on. The luminescent concentrator results in less light absorption immediately under the concentrator; however, a side-on device compensates for this, resulting in a system of two cells that outperforms a single solar cell without a luminescent concentrator. While the efficiency with respect to the projected area was increased, the efficiency of either cell (directly underneath or side-on) was not enhanced. Moreover, such an approach places constraints on the way solar cells are deployed and nullifies the advantage of their flexibility. Also, this approach is less compatible with roll-to-roll processing techniques.

## 4. Exciton Dissociation

**Figure 8:**
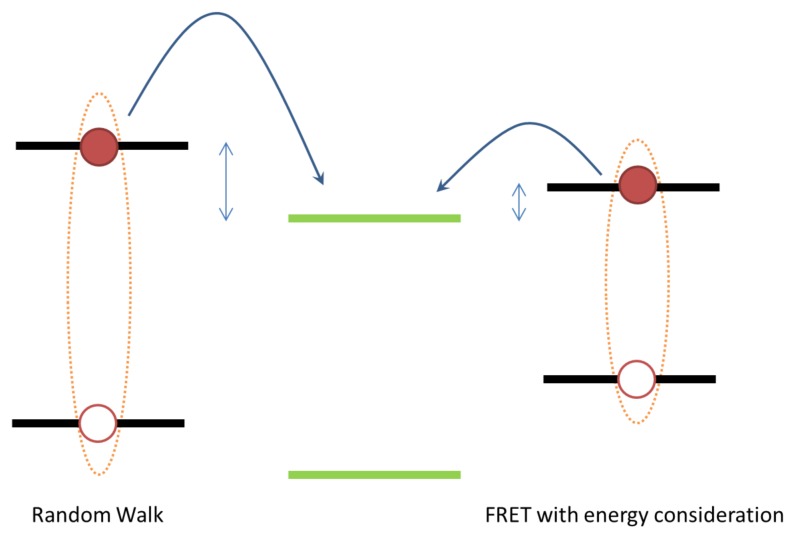
The average energy of an exciton at dissociation in a p-type molecule when (**left**) exciton transport is modelled using a simple random walk and (**right**) energy relaxation is taken into account. The energy levels of an n-type molecule are shown in green. The LUMO offsets are indicated for both cases.

Exciton dissociation may occur through direct charge transfer from the electron/hole conducting moiety to the hole/electron conducting moiety and this process is often treated as the primary dissociation mechanism. The initial dissociation process is driven by the LUMO-LUMO offset (electron injection) and the HOMO-HOMO offset (hole injection) of the two active materials [4]. For example, it has been shown that for certain polymer:fullerene blend systems an energy offset of approximately 0.3 eV is necessary to dissociate the exciton efficiently [112]. Neglecting energy relaxation of the exciton hopping process (as discussed in Section 2) may thus affect the exciton dissociation rate, since the exciton may have insufficient energy to dissociate efficiently (as depicted in Figure 8).

Evidence for a two-step dissociation process has been reported. Lloyd *et al*. [113] proposed a mechanism where excitons in P3HT are transferred to PCBM through FRET, which is followed by direct charge transfer of the hole back into P3HT where it was first created. This process was experimentally observed using an interlayer between electron and hole conducting materials that permits hole transport but not electron transfer. Energy transfer from P3HT to PCBM is certainly possible considering the close correspondence of their band gaps [114] and overlap of the P3HT emission spectrum [115,116] with the PCBM absorption spectrum [113]. This energy transfer process appears to be efficient since facile exciton dissociation was still observed at *~*10 nm barrier thicknesses [117]. Long range FRET has been observed for other hole conducting materials as well [77], highlighting that FRET should not be ignored when modelling exciton diffusion and dissociation.

It should be noted that the two-step dissociation process is only efficient if hole transfer from the electron accepting moiety (e.g., PCBM) to the donating moiety (e.g., P3HT) is facile. While facile hole transfer occurs for many polymers [79,118], this behaviour is not general for all photoactive polymers. For example, poor hole transfer following FRET has been identified for poly[2,8-(6,6,12,12-tetraoctylindenofluorene)-co-4,7-(2,1,3-benzothiodiazole] (IF8BT) due to energy level mismatch [119]. In this case, direct electron transfer drives exciton dissociation and is often less efficient than ultrafast hole transfer from PCBM to the donor. As such, depending on the overlap of photoluminescence and absorption spectra of the photoactive constituents, energy transfer from the donor to the acceptor may or may not be faster than exciton diffusion within the donor followed by direct electron transfer. Hence, the Förster radius directly influences the fraction of excitons that undergo a two-step dissociation process and those that undergo direct charge transfer. Interestingly, very efficient FRET may be a loss mechanism if it is faster than charge transfer at the heterointerface, which is necessary for exciton dissociation [119]. Excitons generated in the fullerene moiety follow diffusion limited one-step dissociation since FRET from fullerenes to polymers is inefficient due to the poor overlap of the respective emission and absorption spectra and the poor fluorescence yield of fullerenes. Hence the domain size of the fullerene is found to be critical in boosting the dissociation efficiency of excitons that are absorbed by the fullerene derivative [120].

Coffey *et al*. [117] argue that optimal BHJ morphologies have domain sizes on length scales that correspond with efficient P3HT:PCBM FRET, suggesting that energy transfer (and not exciton diffusion) dominates exciton dissociation. A direct result of the two-step process is that the band gap of the donor and the acceptor must be similar to maximise emission and absorption spectra overlap and thus enhance FRET. This requirement for spectral overlap may explain the poor dissociation efficiency in some low band gap polymer systems [120] and may also explain the poor dissociation efficiency of polymer/metal oxide interfaces where FRET does not occur [121]. Since fullerenes are commonly used as the electron acceptor, the optimum LUMO level of the donor is constrained by the fullerene LUMO. At the same time, the HOMO of the donor needs to align with the anode to facilitate hole extraction, while a small band gap is desired to maximise light absorption. Energy transfer adds to the complexity of optimising the HOMO and LUMO levels of a material system. All these aspects complicate the search for the ideal material system [17,36].

An interesting corollary of the two-step dissociation process is that it opens up opportunities to add a third photoactive component to the photoactive blend, which normally consists of just two materials (e.g., P3HT:PCBM). Long range FRET ensures exciton dissociation across domain sizes of *~*10 nm. The third component would have to permit hole transfer and may be utilised to cover a larger part of the solar spectrum [117,122].

FRET may also be utilised to draw excitons to the heterointerface where they can be efficiently dissociated. Honda *et al*. [110] used a silicon phthalocyanine derivative (SiPc) as a dye to create ternary devices consisting of P3HT, PCBM and SiPc. The dye extended the spectral absorption range of the device. Their work also indicates that SiPc is selectively located at the P3HT:PCBM interface. Electron transfer from SiPc to PCBM and hole transfer to P3HT is shown to be efficient, thus limiting geminate recombination. Furthermore, the overlap of the photoluminescence with the absorption spectrum of the dye suggests that energy transfer from P3HT to the dye is efficient. Since the dye is located at the heterointerface, it effectively enhances exciton transfer to the heterointerface where efficient dissociation occurs. In this way, Honda *et al*. achieved relative PCE improvements of 20%. They even utilised two dyes giving rise to quaternary blends with which a PCE of 4.3% was achieved. Performance of the quaternary blend was better than either ternary blends (3.7% and 4.1%) or the dye-less P3HT:PCBM blend (3.5%) [108].

## 5. Charge Transfer States and Charge Transport

In organic semiconductors, the photoexcited electron-hole Coulomb interaction is strong owing to their relatively small dielectric constant. As a consequence, CT states (with a Coulomb binding energy larger than *k_B_T*) are generated upon exciton dissociation instead of truly free charge carriers [31]. The energy associated with CT states has been assessed using electroluminescence [37] and absorption measurements (photothermal deflection spectroscopy) [123] and is shown to directly correspond to the HOMO*_donor_*-LUMO*_acceptor_* energy gap, as expected. Understanding CT state dynamics is important as geminate recombination of these states can be a large loss mechanism [124].

Onsager provided a theoretical description of the dissociation dynamics of ion pairs by considering Brownian motion and their Coulomb interaction [38]. In 1984, Braun modified the Onsager model and incorporated the finite lifetime of the CT state in a more realistic manner [30]. Braun also proposed that for disordered organic semiconductors a distribution of nearest neighbour distances exists and thus one has to integrate over this distribution. Further modifications have been proposed as discrepancies between theory and experiment were identified. For example, the Onsager model has been modified to account for high charge mobility [125] and to consider a finite distance-dependent intrinsic reaction rate [126]. Also, the Langevin expression that underpins Onsager theory has been shown not to hold in polymer:fullerene solar cells [127]. So, while the original Onsager theory is relatively easy to implement and provides a theoretical framework to increase the understanding of geminate recombination, its use must be carefully considered for OPV systems.

A modified Onsager model has been incorporated in numerical models that also cover light absorption and charge transport [128,129]. Charge transport dynamics are modelled using the Poisson equation, drift-diffusion equations, and continuity equations in combination with appropriately chosen boundary conditions. These models are often 1D and do not describe the 3D aspect of the nano-scale morphology. In addition, exciton transport is often not considered or is simplified. Despite these simplifications good correspondence is found with experimental data [127,128].

Another approach to modelling CT dynamics is through dynamic Monte Carlo simulations that incorporate charge transfer rate calculation on a molecular scale using Miller-Abrahams expressions [130] and/or Marcus theory [131]. Exciton transport is modelled using either FRET or Dexter theory and the optical field may be determined using transfer matrix techniques [24,105,132]. Monte Carlo simulation captures the effect of Coulomb interaction, induced surface charge at the electrodes, morphology [133–135], electric field (magnitude and direction) [136], energy traps and other energy disorder considerations [24,124]. These models have already proven valuable in understanding OPV operation [133–135,137]. Since Monte Carlo modelling can account for all relevant OPV processes in a 3D fashion starting from basic material properties, it has the potential to provide an all-inclusive model that is able to accurately describe any OPV system [138]. The major disadvantage is the large computational burden associated with Monte Carlo simulations, which makes it less accessible for the broader OPV community and a time consuming approach. Recently, efforts have been focused on still achieving the completeness of dynamic Monte Carlo simulations, but combining it with a computationally faster algorithm to take advantage of the best of both worlds (fast numerical solving of differential equation modelling *vs*. dynamic Monte Carlo modelling) [139].

**Figure 9:**
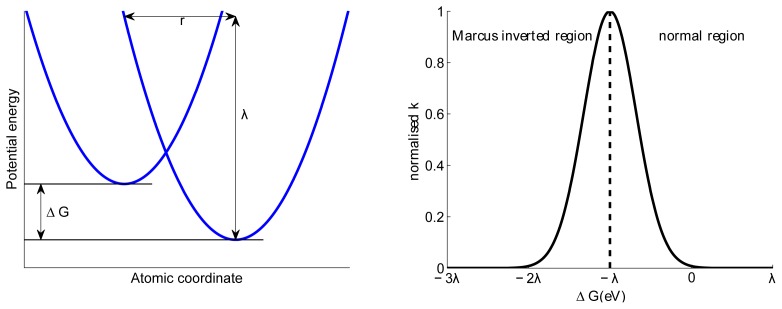
(**a**) Potential energy parabolas of two harmonic oscillators separated by a distance *r*; (**b**) normalised Marcus transfer rate as a function of the free energy difference.

**Figure 10:**
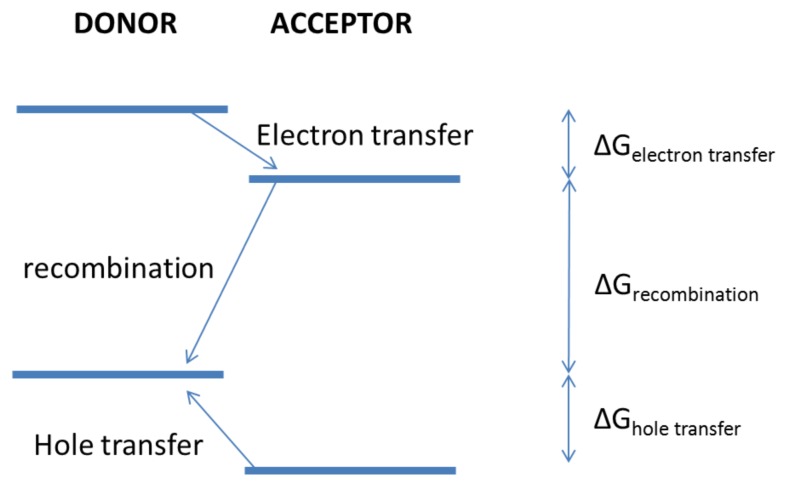
Energy levels associated with donor and acceptor materials.

The use of Marcus theory provides insight into charge (geminate and bimolecular) recombination dynamics. Marcus theory can be used to directly calculate charge recombination rates [140] instead of assigning a fixed recombination rate as is often done in dynamic Monte Carlo simulations. Marcus theory considers the initial and final potential energy surfaces and approximates them by harmonic oscillators; see Figure 9a. Applying Fermis golden rule to determine the rate constant, the Marcus charge hopping/charge recombination rate is given by

(11) $$k=\frac{\pi }{\hbar \sqrt{\pi k_{B}T}}V^{2}e^{-\frac{{\left(\lambda +\Delta G\right)}^{2}}{4\lambda k_{B}T}}$$

where *k_B_* is Boltzmann constant, *T* is temperature, Δ*G* the free energy difference between initial and final state, *λ* the reorganisation energy and *V* the electron coupling term between initial and final states, which decays exponentially with distance [141]. *λ* quantifies the energy required to change the surrounding medium from the equilibrium geometry of the initial state to that of the final state, which is likely to be significant for polarons in organic semiconductors. The most striking aspect of Equation (11) is the quadratic dependence on *λ*+Δ*G*, which gives to the Gaussian dependence of *k* on Δ*G* and allows us to define the so-called inverted region, see Figure 9b. Downward hops are energetically favourable; however, if the energy drop is more than *λ* the associated hopping probability decreases again. This effect has been observed experimentally for many organic materials [142] including derivatives of fullerene and porphyrin electron donor [143]. The optimum free energy difference is exactly *Cλ*. A direct consequence of the inverted region is that geminate (and bimolecular) recombination can be suppressed by employing material systems with a large HOMO*_donor_*-LUMO*_acceptor_* gap [31]. Figure 10 depicts the free energy differences associated with charge transfer and recombination. Since the HOMO*_donor_*-LUMO*_acceptor_* gap is larger than the charge transfer energy offsets, charge recombination essentially occurs in the “inverted” energy region, while charge hopping occurs in the “normal” region. Charge recombination rate constants have been shown to decrease with increasing *λ* and as *|*Δ*G|* becomes smaller [144]. Recently, the Marcus inverted region has been observed for free charge creation in three different polymer:fullerene blends, experimentally establishing the importance of reorganisation energy in OPV devices [145].

Marcus theory predicts that low bandgap polymers will be inherently more prone to charge recombination. Servaites *et al*. [36] concludes that moderate band gap polymers have been more successful than low band gap polymers due to reduced charge recombination in these devices. They reached this conclusion by modelling CT dissociation through a different mechanism known as hot CT dissociation. Here excess energy (below the band gap) is argued to be available and significantly influence the dissociation process [35,146]. The band gap and LUMO offset are taken as important variables, which directly relate to the HOMO*_donor_*-LUMO*_acceptor_* gap. Marcus theory also predicts that charge separation may be greatly improved by decreasing *λ*[140].

## 6. Energy Cascade

Any energy or charge transfer process is influenced by the relative distance and energy levels of the participating reaction centres as evident from energy and charge transfer theories (e.g., FRET and Marcus theory). In order to separate the exciton/charge transfer state efficiently, it is crucial to increase the distance between the corresponding electron and hole. Since Coulomb attraction is high at small distances (0.3–0.7 eV for a dielectric constant in the range 2–4 when charges are 1 nm apart), significant energy is required to increase their relative distance. While charge dissociation within a particular material combination may be inherently inefficient, sequential small steps (distance wise) with high transfer rates may increase the distance between electron and hole and consequently decrease their Coulomb attraction, ultimately leading to fully separated charges.

**Figure 11:**
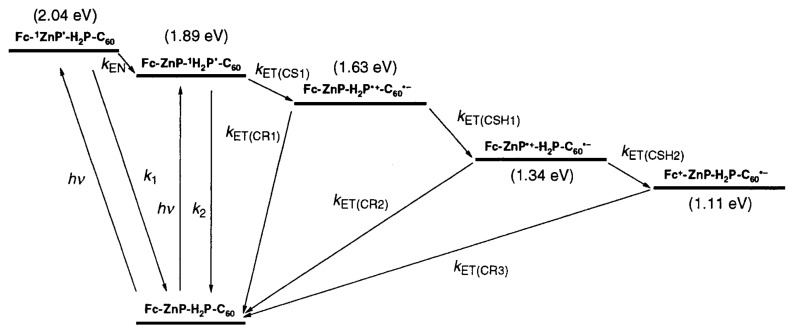
Cascade of energy transfer (*k_EN_*) and electron transfer (*k_ET_* ) leading to an unusually large CT state lifetime of 0.38 s. Reprinted with permission from [32]. Copyright 2001 by The American Chemical Society.

Following Onsager theory, Coulomb interaction may be considered negligible if the corresponding energy is equal to *k_B_T*. Figure 11 depicts a typical energy cascade. Such a process is common in nature (photosynthesis) [31]. Charge separation efficiency of near unity can be achieved through such a relay system, which is likely to compensate for the loss in energy and thus open-circuit voltage due to additional energy losses at each electron transfer step. The reason for this overall gain is that charge recombination is a major loss mechanism in organic solar cells [124]. In fact, since the open-circuit voltage is affected by recombination [147], the addition of a cascade energy relay has been shown to considerably increase the open-circuit voltage for systems with otherwise poor open-circuit voltage [148].

An energy cascade system may provide improvements over standard binary BHJ systems by improving CT-state dissociation through multistep electron transfer [32], but also by funnelling photons through FRET to the appropriate materials [93]. Simultaneous increased light absorption and reduced recombination have been experimentally observed in OPV devices with a cascade donor structure [149]. The cascade devices were shown to perform better than the corresponding single donor reference devices. Depending on energy level alignment, materials can even form an energy cascade for both holes and electrons simultaneously [150]. An energy cascade requires multiple active materials, which makes it challenging to achieve the optimal nano-scale morphology and it is well known that morphology affects all aspects of OPV devices including FRET [151].

Improvements achieved through the addition of interface layers at the electrode-organic interface are often related to improved electron/hole transfer and although it is often not termed as cascade energy alignment, it utilises the same underlying principle that makes natural photosynthetic processes very efficient [152].

## 7. Charge Extraction

Charge extraction is not described by FRET as it does not involve energy transfer. However, in order to provide a complete overview of the entire photoconversion process, we briefly discuss charge extraction. Dissociated charges that reach an electrode have the possibility to be extracted; however a poorly selected electrode interface may prevent this. Several mechanisms are counter-productive towards extracting photogenerated current at the electrode interface including:

- extraction of charges at the opposite polarity electrode (reverse diffusive recombination)
- recombination of charges through defects (e.g., metal penetration into active layer) or impurity states
- formation of an energy barrier due to energy level mismatch of HOMO/LUMO and anode/cathode
- formation of an energy barrier due to electrode corrosion or other degradation effects
- exciton quenching

Metals often have poor charge selectivity, allowing both holes and electrons to be extracted at the same electrode, which consequently lowers photocurrent. Interface layers can be added to improve charge selectivity and alleviate this effect. In fact, all undesired processes at the organic/metal interface may be overcome by using appropriate interface materials [153]. In addition to charge selectivity, interface layers may act as exciton blocking layers, prevent damage of the organic active layer when the top electrode is deposited, prevent chemical reaction with the underlying layer, act as an optical spacer and/or improve energy level alignment [154].

Certain interface layers improve charge extraction by inducing a dipole moment. For instance, LiF is used as an interface layer at the cathode to induce a beneficial dipole moment [155], which changes the effective work function and lowers the energy barrier at the PCBM/Aluminium interface [114]. When charge approaches a perfect metal charge is induced to keep the internal electric field in the metal zero [156]. This induced charge (“image charge”) effectively lowers the potential energy near the electrode interface and is an inherent effect that will always occur.

When the energy barrier at the interface is sufficiently high, charges cannot exit the device at an appreciable rate and thus charge build up occurs. This build-up results in space-charge effects that may give rise to s-shaped current-voltage curves or increased series resistance depending on the severity of the energy barrier [157,158].

## 8. Conclusions

In this review we have discussed the implications of energy transfer in exciton transport, exciton harvesting and exciton dissociation.

FRET describes the transport of singlet excitons, which is often the dominant exciton species in OPV devices. Exceptions are devices based on singlet fission or upconversion. The point dipole approximation fails for polymers or molecules where the conjugation length is much larger than the separation between molecules. In this case, the line-dipole approximation is preferred. Point dipole approximations should also be used with caution for small distances and quantum chemical calculations may be required in certain cases.

Sensitizers may aid in extending the spectral absorption range of organic solar cells and may exhibit both intra and inter-species energy transfer. The addition of a sensitizer to the blend makes controlling morphology, which is an important aspect of binary OPV systems, and understanding interface processes non-trivial unless the system naturally forms a desirable morphology. A separate absorbing layer that gives photoluminescence in the absorption region of the active materials and is not in direct physical contact with the active components may also circumvent this problem. While the addition of a sensitizer has been shown to increase PCE, the concept has yet to be successfully implemented in high efficiency OPV systems.

Energy transfer is the first step of an efficient two-step dissociation process, which requires significant overlap of the PL and absorption spectra of the two photoactive components. However, FRET also competes with charge transfer at the heterointerface (*i.e*., exciton dissociation) turning it into a potential loss mechanism. Energy transfer can also be used to draw excitons to the heterointerface, where efficient exciton dissociation may occur.

An energy cascade comprising both energy transfer and charge transfer may aid in separating charges in OPV devices akin to photosynthetic processes.

Considering the many-fold processes in OPV devices that are influenced by energy transfer, an optimised FRET system offers the prospect of improving OPV performance and consequently aiding in the realisation of plastic solar cells as a low-cost sustainable energy source.
